# Soluble Complement Component 1q Receptor 1 (sCD93) Is Associated with Graft Function in Kidney Transplant Recipients

**DOI:** 10.3390/biom11111623

**Published:** 2021-11-02

**Authors:** Małgorzata Kielar, Paulina Dumnicka, Ewa Ignacak, Alina Będkowska-Prokop, Agnieszka Gala-Błądzińska, Barbara Maziarz, Piotr Ceranowicz, Beata Kuśnierz-Cabala

**Affiliations:** 1 Medical Diagnostic Laboratory with a Bacteriology Laboratory, St. Louis Regional Children’s Hospital, 31-503 Kraków, Poland; gkielar@tlen.pl; 2 Department of Medical Diagnostics, Faculty of Pharmacy, Jagiellonian University Medical College, 30-688 Kraków, Poland; 3 Chair and Department of Nephrology, Faculty of Medicine, Jagiellonian University Medical College, 30-688 Kraków, Poland; ewa.ignacak@uj.edu.pl (E.I.); alina.betkowska-prokop@uj.edu.pl (A.B.-P.); 4 Institute of Medical Sciences, Medical College of Rzeszów University, 35-310 Rzeszów, Poland; agala.edu@gmail.com; 5 Chair of Clinical Biochemistry, Department of Diagnostics, Faculty of Medicine, Jagiellonian University Medical College, 31-066 Kraków, Poland; mbmaziar@cyf-kr.edu.pl (B.M.); beata.kusnierz-cabala@uj.edu.pl (B.K.-C.); 6 Department of Physiology, Faculty of Medicine, Jagiellonian University Medical College, 31-531 Kraków, Poland; piotr.ceranowicz@uj.edu.pl

**Keywords:** kidney allograft, glomerular filtration rate, soluble cluster of differentiation 93, inflammation, albuminuria

## Abstract

Cluster of differentiation 93 (CD93), also known as complement component 1q receptor 1 is a transmembrane glycoprotein expressed in endothelial and hematopoietic cells and associated with phagocytosis, cell adhesion, angiogenesis and inflammation. The extracellular part, soluble CD93 (sCD93), is released to body fluids in inflammation. Data on sCD93 in kidney diseases are limited. Our aim was to evaluate serum sCD93 in long-term kidney transplant recipients as a marker of inflammation and endothelial dysfunction that may be potentially useful in early recognition of graft dysfunction. Seventy-eight adult patients with functioning kidney graft and stable clinical state were examined at least one year after kidney transplantation. Serum sCD93 was measured by enzyme immunosorbent assay. Estimated glomerular filtration rate (eGFR) and albuminuria or proteinuria were assessed at baseline and over one-year follow-up. Increased sCD93 was associated with lower baseline eGFR independently of the confounders. Moreover, sCD93 was negatively associated with eGFR during one-year follow-up in simple analysis; however, this was not confirmed after adjustment for confounders. Baseline sCD93 was positively associated with baseline albuminuria and with increased proteinuria during the follow-up. Serum sCD93 was not correlated with other studied inflammatory markers (interleukin 6, C-reactive protein, procalcitonin and C3 and C4 complement components). To the best of our knowledge, this is the first report regarding the concentrations of sCD93 in kidney transplant recipients and one of the first reports showing the inverse association between sCD93 and renal function. Serum sCD93 should be further evaluated as a diagnostic and prognostic marker in renal transplantation.

## 1. Introduction

Complement component 1q (C1q) receptor 1 (C1qR1, also known as C1qRp, or cluster of differentiation 93—CD93) is a 126-kDa type 1 transmembrane glycoprotein expressed in endothelial and hematopoietic cells, initially described as the receptor for C1q distinct from the 60 kDa calreticulin and responsible for C1q-induced phagocytosis [1,2,3]. The soluble form (sCD93) results from metalloproteinase-dependent shedding of CD93 extracellular domain [4]. The sources of circulating sCD93 include monocytes, neutrophils, and endothelial cells stimulated by inflammatory mediators (tumor necrosis factor α–TNFα and lipopolysaccharide) [4,5,6]. 

CD93 has been identified as a receptor for three structurally similar proteins inducing phagocytosis, namely C1q, mannose-binding lectin and pulmonary surfactant protein A [2]. Although McGreal et al. [7] have shown that the protein does not bind C1q in physiological state, novel studies have confirmed the association of CD93 with phagocytosis of apoptotic cells [8,9]. In addition, CD93 has been shown to be involved in angiogenesis, endothelial cell adhesion, and inflammatory response [4,5,10,11]. Increased expression of membrane CD93 was observed on activated macrophages along with increased concentrations of sCD93 in plasma [5]. Consequently, increased circulating sCD93 was observed in acute and chronic inflammatory conditions in human, including myocardial infarction [12], autoimmune [13,14,15,16] and allergic diseases [17,18,19].

There are single reports linking sCD93 with renal diseases, i.e., diabetic nephropathy in type 2 diabetes [6], and renal involvement in antineutrophil cytoplasmic antibody associated vasculitis [16]. In 2016, Ikewaki et al. [20] reported a strong correlation between sCD93 and serum creatinine and cystatin C in 14 patients with chronic kidney failure. However, to our best knowledge, sCD93 has not been studied in kidney transplant recipients.

Renal transplantation is currently the best therapeutic option in end-stage kidney failure, enabling considerably better quality of life and decreased morbidity and mortality as compared to other renal replacement therapies (dialysis). Nonetheless, a range of inflammatory conditions including acute or chronic rejection, recurrent or de novo nephropathies, cardiovascular complications or infections are associated with limited survival of kidney grafts and kidney transplant recipients [21]. In clinical routine, the monitoring of kidney graft function is still based on periodic physical examination of the recipient, the assessment of urine volume, serum creatinine, calculated (estimated) glomerular filtration rate (eGFR) and albuminuria / proteinuria, with kidney graft biopsy reserved to clinically relevant situations of decreasing graft function [22]. However, in many cases, increased serum creatinine and albuminuria may be late signs of disease processes affecting the kidney graft [23]. There is a need for novel biomarkers that may serve as early warning signs of transplant injury, enabling faster diagnosis and early treatment [24].

The aim of our study was to assess serum sCD93 concentrations in patients with long-term functioning kidney transplant in relation to other inflammatory markers and the longitudinal changes in transplant function.

## 2. Materials and Methods

### 2.1. Patients and Study Protocol

The prospective observational study included ambulatory patients of kidney transplant recipients’ ambulatory, Chair and Department of Nephrology, University Hospital, Kraków, Poland between May and July 2019. The study included adult patients (at least 18 years of age) who had received the kidney transplant at least one year before the start of the study, and had the functioning kidney transplant at the start of the study (eGFR at least 15 mL/min/1.73 m^2^). The exclusion criteria were: acute kidney injury defined according the KDIGO 2011 [25], any condition requiring hospital treatment during three months before the recruitment, and signs or symptoms of any infection (including urinary tract infection) at the time of recruitment. The present study included a subset of a larger group described previously [26]; the measurements of serum sCD93 were performed in the sera of the first 78 patients recruited.

The study protocol was approved by the Jagiellonian University Bioethical Committee (approval no 1072.6120.46.2019 issued on 28 February 2019). On recruitment, patients signed an informed consent for the study. 

Patients were recruited by the experienced nephrologist during their control ambulatory visit in kidney transplant recipients’ ambulatory, following the detailed history and physical examination. For the purposes of the study, the clinical data at baseline were recorded, including immunosuppressive therapy, comorbidities, date of transplantation, deceased or living donor, first or second transplant, induction therapy, cold and warm ischemia time, delayed graft function and the primary cause of kidney disease based on the available medical records. Moreover, data on pretransplant panel reactive antibodies (PRA) and donor/recipient human leukocyte antigens (HLA) mismatches based on transplantation protocols were collected when available; however, these were available only in cases who had undergone the transplantation procedure in the study center (University Hospital, Kraków, Poland). 

The follow-up data were recorded in September 2020, i.e., after 12–14 months after patient recruitment and included the medical records on graft function during the follow-up and the causes of graft dysfunction, all available serum creatinine concentrations measured during the follow-up, and the results of laboratory tests performed at the last visit of the patient to the kidney transplant recipients’ ambulatory ward.

### 2.2. Laboratory Tests

Fasting urine and venous blood samples for the laboratory tests described below were collected in the morning of the day of the recruitment. 

The routine laboratory tests were performed on the day of blood collection in the Department of Diagnostics, University Hospital, Kraków, Poland, with the use of automated analyzers (Sysmex XN 2000 hematology analyzer, Sysmex Corporation, Cobe, Japan and Cobas PRO Roche Diagnostics, Mannheim, Germany). These included complete blood count, serum creatinine, albumin, glucose, triglycerides, total cholesterol, C-reactive protein (CRP), urine albumin and creatinine, and the concentrations of immunosuppressive drugs (cyclosporine and tacrolimus). Urine albumin-to-creatinine ratio (ACR) was calculated by dividing the respective concentrations. Estimated GFR was calculated based on serum creatinine, age, sex and race using the Chronic Kidney Disease Epidemiological Collaboration (CKD-EPI) 2009 formula [27]. 

For the purpose of the study, additional tests were performed using automated analyzers and dedicated reagent kits: serum concentrations of procalcitonin and interleukin 6 were measured using Cobas 6000 (Roche Diagnostics, Mannheim, Germany), and serum concentrations of complement components 3 (C3) and 4 (C4) were measured using BN II nephelometer (Siemens Healthcare, Erlangen, Germany). 

The excess of serum samples collected for routine tests were aliquoted and frozen in −80 °C for additional tests. Serum concentrations of sCD93 was measured in a series of samples using Quantikine ELISA Human C1qR1/CD93 Immunoassay (R&D Systems, McKinley Place, MN, USA) and the microplate reader Micro ELISA Reader ELX 808 (BIO-TEK Instruments Inc., Winooski, VT, USA). The measurements were performed immediately after collection of all samples. Samples were analyzed in duplicate. According to the manufacturer’s data, the minimum detectable dose of human sCD93 ranged from 0.001 to 0.028 ng/mL (mean 0.006 ng/mL) and the serum concentrations in healthy individuals ranged from 90.0 to 223.0 ng/mL (mean 146.0 ng/mL). The measurements were performed at the Department of Diagnostics, Chair of Clinical Biochemistry, Jagiellonian University Medical College, Krakow, Poland.

### 2.3. Statistical Analysis

Categorical data were presented as number of patients and percentage of the studied group. Quantitative data were presented as mean ± standard deviation (SD) for normally distributed variables and median, lower quartile (Q1) and upper quartile (Q3) for non-normally distributed variables. The distributions were assessed with Shapiro–Wilk’s test. Because serum concentrations of sCD93 and other studied inflammatory markers were non-normally distributed, we compared them between subgroups (specified in Results) using non-parametric tests (Mann–Whitney or Kruskal–Wallis, depending on the number of subgroups). The baseline and follow-up data were compared using paired t-test or Wilcoxon matched pairs test, according to distribution. Simple correlations were analyzed with Pearson’s coefficient and univariate linear regression, after log-transformation of right skewed variables (including sCD93). Multiple linear regression was calculated using the independent variables that were associated with the outcome variable in simple analysis at *p* < 0.1. We reported standardized regression coefficients (β) with standardized errors (SE). The multiple linear regression models were adjusted for clinically relevant covariates, i.e., age, time from transplantation, sex, and diabetes, as shown in Results. The results were considered significant at *p *< 0.05. Statistica 13.3 software (TIBCO Software Inc., Tulsa, OK, USA) was used for computations.

## 3. Results

### 3.1. Clinical Characteristics of Studied Kidney Transplant Recipients

The study included 78 patients aged between 26 and 78 years, and between 1 and 22 years post renal transplantation (Table 1). The stages of chronic kidney disease assessed according to 2012 KDIGO guidelines [27] and based on baseline eGFR and urine ACR were as follows: G1T in 4 (5%), G2T in 22 (28%), G3aT in 15 (19%), G3bT in 29 (37%), G4T in 8 (10%), A1 in 36 (46%), A2 in 26 (33%), and A3 in 16 (21%) patients. All the study participants were ambulatory patients of the post-transplant ambulatory ward of a tertiary center. 

The baseline results of laboratory tests in the studied group (Table 2) were within the reference ranges in most patients, with the exception of increased urine albumin, ACR, and serum creatinine, low eGFR, and low blood hemoglobin, which represent the abnormalities typical for chronic kidney disease. Serum concentrations of sCD93 were above 223 ng/mL (i.e., the maximum value observed in healthy individuals as reported by the manufacturer of the test) in 59 patients (76% of the studied group). On the contrary, serum CRP exceeded the upper reference limit of 3 mg/L in 20 patients (26%), serum interleukin 6 was above the upper reference limit in 26 patients (33%), and there were no patients with increased procalcitonin. Single patients presented with serum C3 and C4 concentrations below (five patients, 6%, and one patient, 1%, respectively) or above (two patients, 3%, and seven patients, 9%, respectively) the reference range.

### 3.2. The Associations between sCD93, Other Studied Inflammatory Markers and the Baseline Characteristics of Patients

**Figure 1:**
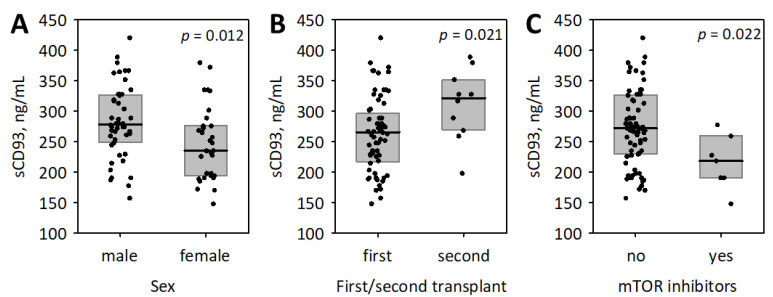
The association between serum concentrations of cluster of differentiation 93 (sCD93) and the baseline clinical characteristics of studied kidney transplant recipients: sex (**A**), first or second transplant (**B**), and the treatment with mammalian target of rapamycin (mTOR) inhibitors (**C**). Data are shown as median (line), interquartile range (box), and raw data (points); *p*-values in Mann–Whitney test are presented.

Serum sCD93 concentrations were higher among men (Figure 1). We did not observe significant associations between log (sCD93) and age (R = −0.18; *p* = 0.1) or log-transformed time from transplantation (R = 0.13; *p* = 0.3). The second transplant recipients had significantly higher serum sCD93 concentrations as compared with the patients after first transplantation (Figure 1). sCD93 concentrations did not differ in patients with various categories of underlying kidney disease (glomerular, tubulointerstitial, vascular, congenital or unknown; *p* = 0.2) and between patients with or without diabetes (*p* = 0.8). Patients treated with mTOR inhibitors were characterized with significantly lower concentrations of sCD93 (Figure 1), while we observed no other associations with immunosuppressive treatment. Additionally, there were no significant correlations between log (sCD93) and the measured concentrations of immunosuppressive medications (tacrolimus: R = 0.12; *p* = 0.4; cyclosporine: R = −0.19; *p* = 0.4). There was a weak correlation between log (sCD93) and systolic blood pressure (R = 0.26; *p* = 0.028) and no correlation with diastolic blood pressure (R = 0.05; *p* = 0.7).

In the subset of patients with available data on the transplantation procedure, the concentrations of sCD93 (log-transformed) were not correlated with the number of mismatched HLA (R = 0.33; *p* = 0.1) nor with the maximum (R = 0.21; *p* = 0.3) or last pretransplant PRA (R = 0.07; *p* = 0.7). Additionally, the sCD93 concentrations were not associated with cold (R = 0.21; *p* = 0.1) or warm ischemia time (R = 0.10; *p* = 0.5), the use of induction therapy (median 271 versus 262 ng/mL; *p* = 0.5), or the delayed graft function (median 275 versus 257 ng/mL; *p* = 0.064).

We observed no significant correlations between the studied serum concentrations of inflammatory markers or complement components and sCD93 (*p* > 0.1; Table 3). Baseline serum creatinine, eGFR, urine albumin and urine ACR significantly correlated with sCD93 concentrations. In multiple regression, baseline eGFR, urine ACR, male sex, second transplantation, and treatment with mTOR inhibitors were all significantly and independently associated with serum sCD93 (Table 3).

### 3.3. The Associations between Studied Inflammatory Markers and Follow-Up Data

After a 12-month follow-up (median length 12.7; Q1; Q3: 11.2; 13.4; range: 3.2; 15.4 months), serum creatinine concentrations (median: 117; Q1: 97; Q3: 164 µmol/L) and eGFR values (median: 51; Q1: 36; Q3: 71 mL/min/1.73 m^2^) in the studied group of kidney transplant recipients did not differ significantly compared to the initial values (*p* = 0.8 for both variables). Additionally, the mean serum creatinine calculated as the arithmetic mean of all measurements available in the follow-up period (median: 121; Q1: 100; Q3: 164 µmol/L) and the eGFR based on mean creatinine (median: 50; Q1: 36; Q3: 69 mL/min/1.73 m^2^) did not differ significantly from the initial values (*p* = 0.7 and *p* = 0.9, respectively). There was one patient in whom serum creatinine doubled during the follow-up; two started dialysis. During the follow-up period, the attending physicians noted transient decrease in kidney graft function in 14 (18%) patients (due to general infection in three patients, urinary tract infection in two, adverse drug events in one and cardiovascular complication in one patient; unknown cause in seven patients), and persistent decrease in graft function in eight (10%) patients (caused by graft rejection in two patients, recurrence of glomerulonephritis in one, and urological complications in one; unknown cause in four, who rejected the graft biopsy). The increased proteinuria (A2 or A3 according to KDIGO [27]) at the end of follow-up was observed in 16 patients (21%) of whom two were diagnosed with graft rejection, one with recurrent glomerulonephritis, four with urinary tract infections, one with urological complications, and one with monoclonal gammopathy; the cause was not known in the remaining seven cases. Twelve patients (15%) developed urinary tract infection during the follow-up period.

Baseline serum sCD93 concentrations (log-transformed) correlated significantly with mean and final eGFR during the follow-up (Table 4 and Table 5). Other significant predictors of mean (Table 4) and final eGFR (Table 5) in simple analysis included baseline eGFR, time from transplantation, urine albumin and ACR, serum total cholesterol and triglycerides, serum interleukin 6 and serum procalcitonin. In multiple regression including all significant univariate predictors, serum sCD93 concentration was not significantly associated with mean and final eGFR independently of the covariates (Table 4 and Table 5, multiple model 1). Because the correlations between the baseline, mean and final eGFR were very strong, and similarly, there was a strong correlation between baseline serum cholesterol and triglycerides, we also calculated additional regression models excluding baseline eGFR and baseline triglycerides (Table 4 and Table 5, multiple model 2). In such models, sCD93 concentrations were associated with eGFR during the follow-up independently of the remaining predictors.

**Figure 2:**
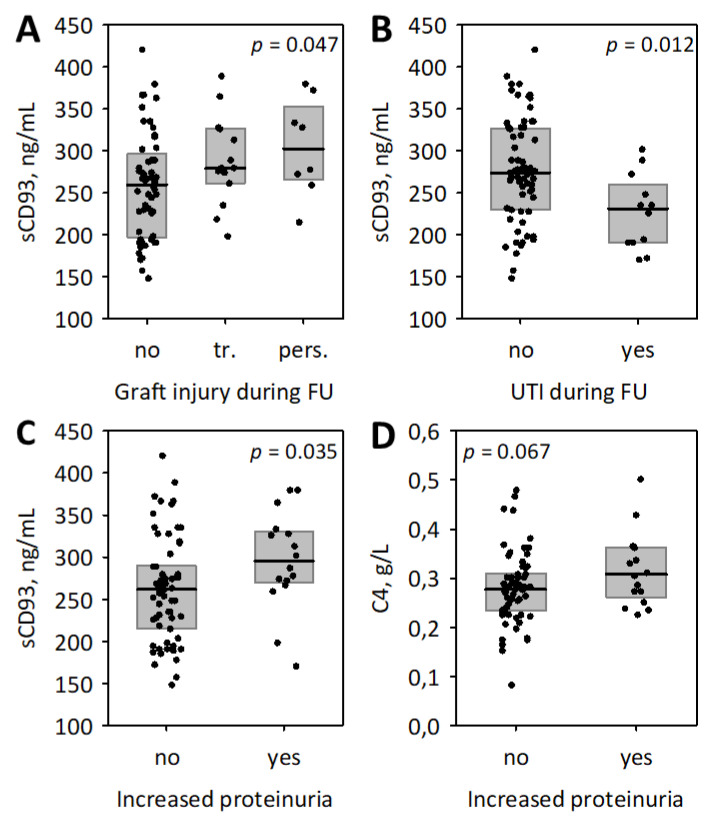
The associations between baseline serum sCD93 and other studied inflammatory markers and the follow-up (FU) data: the association of sCD93 with the clinically significant transient (tr.) or persistent (pers.) graft injury (**A**); the association of sCD93 and urinary tract infection (UTI) during the follow-up (**B**); the associations of sCD93 (**C**) and complement component 4 (C4) (**D**) with increased proteinuria during the follow-up (i.e., at least A2 proteinuria that persisted or developed during the follow-up and was present at the end of observation). Data are shown as median (line), interquartile range (box), and raw data (points); *p*-values obtained using Kruskal–Wallis (**A**) and Mann–Whitney (**B**–**D**) test are presented.

Serum concentrations of sCD93 were significantly positively associated with the adverse renal events during the follow-up, i.e., transient or persistent decrease in graft function recorded by the post-transplant ambulatory physician, and increased or persistent proteinuria (Figure 2). This was despite lower sCD93 concentrations being observed in patients who experienced urinary tract infections during the follow-up (Figure 2B). Of the studied inflammatory markers, higher baseline serum C4 was associated with the increased proteinuria (Figure 2D).

## 4. Discussion

The main finding of our study is the correlation of serum sCD93 with kidney graft function observed in long-term kidney transplant recipients examined at least one year following the transplantation procedure. Increased sCD93 was associated with lower baseline eGFR independently of the confounders. Increased sCD93 was associated with lower eGFR during one-year follow-up, although we were not able to confirm the association after adjustment for covariates. Moreover, sCD93 was positively associated with urine albumin/creatinine ratio at baseline and with increased proteinuria during the follow-up. Of note, sCD93 was not correlated with other studied inflammatory markers, and the observed relationships between interleukin 6, procalcitonin, or C4 and kidney function were weaker and less consistent than the relationships between sCD93 and kidney function. To the best of our knowledge, this is the first report regarding the concentrations of sCD93 in kidney transplant recipients.

The observed association between sCD93 and kidney function is consistent with several previous reports. Lee et al. [6] measured sCD93 in 97 adult patients with type 2 diabetes. Those with sCD93 above median were characterized by significantly lower eGFR, and higher prevalence of diabetic nephropathy and A2 albuminuria (urine ACR >300 mg/g) [6]. The authors [6] observed a significant positive correlation between urine albumin/creatinine ratio and serum sCD93, similarly to our observations. On the other hand, the negative correlation between sCD93 and eGFR was weaker as compared to our findings, which may be explained by a small diversity in eGFR in the study of Lee et al. [6], which included only single patients with eGFR <60 mL/min/1.73 m^2^ while our patients represented a broader range of eGFR. Furthermore, Ikewaki et al. [20] showed that sCD93 was increased in 14 patients with chronic kidney failure compared to 10 healthy controls, and that serum sCD93 concentrations in patients were strongly positively correlated with blood urea nitrogen, serum creatinine and cystatin C. Unfortunately, these 14 chronic kidney failure patients are very poorly characterized in the report [20]. In Japanese patients with antineutrophil cytoplasmic antibody associated vasculitis, renal involvement and active renal disease were associated with higher serum sCD93 concentrations, and sCD93 negatively correlated with eGFR, with concentrations above 356 ng/mL being indicative for end-stage renal disease [16]. Finally, Mälarstig et al. [28] observed a positive correlation between sCD93 and cystatin C but no correlation with eGFR in a cohort of patients with a history of myocardial infarction and healthy controls (a total of 768 participants). However, in this latter study [28], the vast majority of patients had good renal function (the lower quartile of eGFR was 70 mL/min). In addition, CD93 has been reported to be expressed in kidney endothelial cells, both glomerular and interstitial [29]. In diabetic mice, glomerular endothelial cells were characterized by enhanced expression of membrane CD93, and sCD93 was shed from these cells, resulting in increased sCD93 concentrations in peritoneal fluid and urine, along with the development of albuminuria and histological changes in the glomeruli, indicative for diabetic nephropathy [6].

The negative association between sCD93 and glomerular filtration could be attributed to renal retention of sCD93. However, sCD93 is not a low-molecular-mass protein. Greenlee et al. [5] observed that the extracellular CD93 fragments of 50-75 kDa were shed from activated peritoneal macrophages under inflammatory conditions. Similarly, Bohlson et al. [4] detected 50 kDa and 75 kDa fragments shed from monocytes stimulated with phorbol ester. These molecular masses are more comparable to the molecular mass of albumin (69 kDa), rather than the range of freely filtered low-molecular-weight proteins used to (or proposed to) assess glomerular filtration (e.g., cystatin C, 13 kDa; beta-trace protein, 18.5 kDa, beta2-microglobulin, 12 kDa; or retinol-binding protein, 21 kDa) [30]. Therefore, it does not seem likely that the association between sCD93 and kidney transplant function in our patients is solely due to renal retention of the protein.

The shedding of CD93 from cells is dependent on the degree of O-glycosylation of the protein [10]. Thus, the higher concentrations of sCD93 might perhaps be partially explained by decreased or altered glycosylation of CD93 attributed to dysregulated glycosylation in uremic milieu [20]. Genetic abnormalities associated with the deficit of glycosyltransferase Galnt11 has been associated with chronic kidney disease [31]. However, this explanation remains speculative.

Increased sCD93 may also be a sign of chronic inflammation and endothelial dysfunction observed in patients with chronic kidney disease, including kidney transplant recipients. Chronic inflammation in renal insufficiency may be due to several causes, including increased production and renal retention of proinflammatory cytokines, endothelial dysfunction induced by uremic toxins, or enhanced oxidative stress [32,33,34,35]. Increased serum or plasma concentrations of inflammatory markers, including CRP, interleukin 6 and TNFα are consistently reported in patients with chronic kidney disease [34,36,37]. Moreover, these inflammatory markers negatively correlate with GFR in patients with chronic kidney disease [36]. In a large cohort of patients with chronic kidney disease (the Chronic Renal Insufficiency Cohort—CRIC—study), faster progression of kidney insufficiency was shown to be positively associated with chronic inflammation and increased inflammatory markers, including interleukin 6 and TNFα [34]. Cottone et al. [38] and Małyszko et al. [39] have shown increased plasma concentrations of TNFα and laboratory markers of endothelial activation or dysfunction (intercellular and vascular cell adhesion molecules, CD44, CD146, thrombomodulin, von Willebrand factor and others) in long-term renal transplant recipients with similar characteristics to our patients. In kidney transplant recipients, the concentrations of TNFα, intercellular and vascular cell adhesion molecule in plasma were all negatively correlated with eGFR, indicating vascular inflammation and endothelial activation aggravated in patients with decreased eGFR [38]. These findings are in line with ours, considering that sCD93 is shed from endothelial cells and TNFα has been shown to induce the shedding [4,5,6]. However, it must be acknowledged that we did not observe significant correlations between sCD93 and other studied inflammatory markers: interleukin 6, CRP, procalcitonin, and complement components C3 and C4; and that we did not measure TNFα. The distinct inflammatory markers are known to be variably increased in chronic kidney disease [37]. Although we observed the associations between interleukin 6 or procalcitonin and kidney function in studied kidney transplant recipients, the associations were weaker and less consistent than that between sCD93 and kidney graft function. 

Interestingly, we observed lower serum sCD93 in patients who developed urinary tract infections during the follow-up than in those who did not. In the study of Ishizaki et al. [16], sCD93 concentrations were higher in patients with antineutrophil cytoplasmic antibody associated vasculitis as compared to those with bacterial infections. This may support the hypothesis that sCD93 is differentially regulated depending on the type and the cause of inflammation. This would explain the lack of significant correlation between sCD93 and CRP or procalcitonin (the markers that most significantly increase in bacterial infections).

In kidney transplant recipients, treatment with immunosuppressive drugs and corticosteroids may influence the concentrations of sCD93. Although we only observed lower concentrations of the protein in patients receiving mTOR inhibitors, we cannot exclude other relationships, because the immunosuppressive regimens were similar in the majority of our patients. Treatment with inhaled corticosteroids was reported to decrease serum concentrations of sCD93 in allergic diseases [18]. In our study group, 96% of patients received oral glucocorticoids.

Our study has several limitations; therefore, our results regarding serum sCD93 as a predictor of renal transplant function should be considered preliminary. First, we included limited number of patients and the follow-up time was short. For that reason, we were not able to determine the association between sCD93 with such endpoints as doubling serum creatinine, progression to end-stage kidney failure, or mortality (including cardiovascular mortality), as these endpoints were observed in single patients. Moreover, our report provides incomplete characteristics of patients at the time of transplantation (due to limited availability of data and long time from transplantation in many patients), and incomplete data about the causes of decline in renal graft function observed during follow-up. Larger studies with longer follow-up and preferably with the use of protocol biopsies are needed to confirm and explore the diagnostic and prognostic usefulness of sCD93 in kidney transplant recipients. Experimental studies are needed to explain the mechanism of sCD93 increase in chronic kidney disease. Nonetheless, our results indicate that renal function should be considered as a confounder in epidemiological studies evaluating sCD93 as a marker of diseases.

## 5. Conclusions

In summary, although based on limited number of studied patients, this is one of the first reports showing the association between serum sCD93 and kidney function, and the first such observation in kidney transplant recipients. In long-term renal transplant recipients, soluble CD93 positively correlated with serum creatinine and urine albumin/creatinine ratio and negatively correlated with eGFR. The association between sCD93 and kidney function should be considered in future studies evaluating sCD93 as a marker of inflammation or endothelial dysfunction. Larger studies with longer follow-up are required to confirm the usefulness of CD93 as a diagnostic and prognostic marker indicating graft dysfunction in kidney transplant recipients. 

## Figures and Tables

**Table 1:** Baseline clinical characteristics of studied group of 78 kidney transplant recipients.

Characteristic	Values
Mean age ± SD, years	53 ± 13
Male sex, *n* (%)	47 (60)
Median time from transplantation (Q1; Q3), years	8.0 (5.0; 15.0)
Primary cause of kidney disease	
Glomerular diseases, *n* (%)	29 (37)
Tubulointerstitial diseases, *n* (%)	10 (13)
Vascular diseases, *n* (%)	3 (4)
Cystic/congenital diseases, *n* (%)	10 (13)
Unknown, *n* (%)	26 (33)
First transplant, *n* (%)	68 (87)
Second transplant, *n* (%)	10 (13)
Deceased donor, *n* (%)	77 (99)
Induction therapy, *n* (%)	8 (10)
No data, *n* (%)	23 (29)
Median cold ischemia time (Q1; Q3), min	1200 (840; 1500)
No data, *n* (%)	19 (24)
Median warm ischemia time (Q1; Q3), min	31 (26; 40)
No data, *n* (%)	19 (24)
Median number of donor-recipient HLA mismatches (Q1; Q3)	3 (3; 4)
No data, *n* (%)	52 (67)
Median peak pretransplant PRA (Q1; Q3), %	0 (0; 3)
Maximum peak pretransplant PRA, %	50
No data, *n* (%)	52 (67)
Median last pretransplant PRA (Q1; Q3), %	0 (0; 0)
Maximum last pretransplant PRA, %	50
No data, *n* (%)	52 (67)
Delayed graft function, n (%)	21 (27)
No data, *n* (%)	18 (23)
Immunosuppressive therapy	
glucocorticoids, *n* (%)	75 (96)
MMF or MPA, *n *(%)	73 (94)
tacrolimus, *n* (%)	48 (62)
cyclosporine, *n* (%)	24 (31)
mTOR inhibitor, *n* (%)	7 (9)
Diabetes, *n* (%)	13 (17)
Hypoglycemic agents	
oral, *n* (%)	10 (13)
insulin, *n* (%)	5 (6)
Median daily diuresis (Q1; Q3), L	2500 (2000; 3000)
Mean BMI ± SD, kg/m^2^	26.9 ± 4.9
Mean systolic pressure ± SD, mmHg	133.9 ± 15.0
Mean diastolic pressure ± SD, mmHg	83.8 ± 10.7

SD, standard deviation, Q1, lower quartile; Q3, upper quartile; HLA, human leukocyte antigens; PRA, panel reactive antibodies; MMF, mycophenolate mofetil; MPA, mycophenolic acid; mTOR, mammalian target of rapamycin; BMI, body mass index.

**Table 2:** The results of selected laboratory tests in the studied group of 78 kidney transplant recipients at the start of the study. Data are shown as mean ± standard deviation or median (lower; upper quartile).

Laboratory Test	Results	Reference Range
Urine albumin, mg/L	28.5 (7.0; 200.0)	<20
Urine albumin/creatinine ratio, mg/g	39.3 (10.2; 222.1)	<30
Serum creatinine, µmol/L	128 (92; 168)	F: 44–80; M: 62–106
eGFR, mL/min/1.73 m^2^	47 (36; 71)	>60
Hemoglobin, g/dL	13.2 ± 1.7	F: 12.0–16.0; M: 14.0–18.0
White blood cell count, ×10^3^/µL	7.36 (5.85; 8.42)	4.5–10.0
Triglycerides, mmol/L	1.67 (1.22; 2.14)	<2.26
Total cholesterol, mmol/L	5.01 (4.41; 5.61)	3.50–5.20
Glucose, mmol/L	5.53 (5.15; 6.02)	3.30–5.60
Serum albumin, g/L	44 (42; 46)	35–52
C-reactive protein, mg/L	1.51 (1.00; 3.31)	<3.0
Procalcitonin, ng/mL	0.061 (0.044; 0.095)	<0.5
Interleukin 6, pg/mL	4.71 (2.51; 7.63)	<7.0
C3, g/L	1.22 (1.10; 1.36)	0.9–1.8
C4, g/L	0.280 (0.236; 0.323)	0.1–0.4
sCD93, ng/mL	269 (227; 316)	90–223 *

* the minimum–maximum in healthy individuals as reported by the manufacturer of the test; eGFR, estimated glomerular filtration rate; sCD93, soluble cluster of differentiation 93; C3, complement component 3; C4, complement component 4.

**Table 3:** Simple (univariate) and multiple regression showing the variables independently associated with log-transformed serum sCD93 concentration as the dependent variable. The clinical and laboratory data obtained at the start of the study were used to construct the regression models.

Independent Variable	Simple Regression		Multiple Regression	
	β ± SE	* p *	β ± SE	* p *
log (interleukin 6)	−0.11 ± 0.11	0.3	not included	
log (C-reactive protein)	–0.14 ± 0.11	0.2	not included	
log (procalcitonin)	0.17 ± 0.11	0.13	not included	
log (C3)	−0.08 ± 0.12	0.5	not included	
log (C4)	0.07 ± 0.12	0.5	not included	
log (serum creatinine)	0.61 ± 0.09	<0.001	not included	
eGFR	−0.51 ± 0.10	<0.001	−0.47 ± 0.10	<0.001
log (urine albumin)	0.34 ± 0.11	0.002	not included	
log (urine ACR)	0.37 ± 0.11	0.001	0.22 ± 0.11	0.040
Age	−0.18 ± 0.11	0.11	−0.11 ± 0.10	0.3
log (time from transplantation)	0.13 ± 0.11	0.3	0.05 ± 0.09	0.6
Male sex	0.29 ± 0.11	0.009	0.20 ± 0.10	0.043
Diabetes	−0.04 ± 0.11	0.8	−0.04 ± 0.09	0.6
Second transplant	0.25 ± 0.11	0.026	0.22 ± 0.09	0.019
Treatment with mTOR inhibitors	−0.27 ± 0.11	0.015	−0.31 ± 0.09	0.001
Systolic blood pressure	0.26 ± 0.12	0.028	−0.02 ± 0.10	0.8
Whole model	not applicable		R^2^ = 0.55	*p* < 0.001

ACR, albumin/creatinine ratio; SE, standard error.

**Table 4:** Simple (univariate) and multiple regression models using baseline data to predict eGFR based on mean serum creatinine during the follow-up (mean eGFR).

Independent Variable	Simple Regression		Multiple Model 1		Multiple Model 2	
	β ± SE	* p *	β ± SE	* p *	β ± SE	* p *
Baseline eGFR	0.92 ± 0.05	<0.001	0.84 ± 0.06	<0.001	not included	
log (urine ACR)	−0.37 ± 0.11	<0.001	−0.11 ± 0.05	0.038	−0.20 ± 0.10	0.057
Total cholesterol	−0.29 ± 0.11	0.010	0.05 ± 0.05	0.3	−0.16 ± 0.10	0.10
log (triglycerides)	−0.28 ± 0.11	0.015	−0.03 ± 0.05	0.6	not included	
log (interleukin 6)	−0.26 ± 0.11	0.022	−0.12 ± 0.06	0.053	−0.28 ± 0.12	0.019
log (procalcitonin)	−0.29 ± 0.11	0.010	0.05 ± 0.06	0.4	−0.002 ± 0.12	1.0
log (sCD93)	−0.50 ± 0.10	<0.001	−0.04 ± 0.06	0.5	−0.40 ± 0.11	<0.001
Age	−0.13 ± 0.11	0.3	−0.03 ± 0.05	0.5	−0.17 ± 0.09	0.08
log (time from Tx)	−0.23 ± 0.11	0.047	−0.04 ± 0.05	0.4	−0.12 ± 0.09	0.19
Male sex	−0.13 ± 0.11	0.3	0.02 ± 0.05	0.7	−0.11 ± 0.10	0.2
Diabetes	−0.16 ± 0.11	0.15	−0.08 ± 0.05	0.09	−0.13 ± 0.09	0.2
Whole model	not applicable		R^2^ = 0.93	*p* < 0.001	R^2^ = 0.50	*p* < 0.001

Tx, transplantation.

**Table 5:** Simple (univariate) and multiple regression models using baseline data to predict final eGFR at the end of follow-up.

Independent Variable	Simple Regression		Multiple Model 1		Multiple Model 2	
	β ± SE	* p *	β ± SE	* p *	β ± SE	* p *
Baseline eGFR	0.91 ± 0.05	<0.001	0.81 ± 0.07	<0.001	not included	
log (urine ACR)	−0.36 ± 0.11	0.002	−0.08 ± 0.06	0.2	−0.20 ± 0.10	0.050
Total cholesterol	−0.32 ± 0.11	0.005	−0.01 ± 0.06	0.9	−0.20 ± 0.09	0.035
log (triglycerides)	−0.26 ± 0.11	0.020	−0.004 ± 0.05	0.9	not included	
log (interleukin 6)	−0.24 ± 0.11	0.033	−0.09 ± 0.07	0.2	−0.25 ± 0.12	0.040
log (procalcitonin)	−0.30 ± 0.11	0.007	−0.001 ± 0.07	1.0	−0.08 ± 0.12	0.7
log (sCD93)	−0.52 ± 0.10	<0.001	−0.06 ± 0.07	0.4	−0.40 ± 0.11	<0.001
Age	−0.13 ± 0.11	0.3	−0.004 ± 0.06	0.9	−0.14 ± 0.09	0.14
log (time from Tx)	−0.24 ± 0.11	0.037	−0.06 ± 0.05	0.2	−0.14 ± 0.09	0.14
Male sex	−0.18 ± 0.11	0.13	−0.02 ± 0.06	0.7	−0.15 ± 0.10	0.13
Diabetes	−0.12 ± 0.11	0.3	−0.05 ± 0.05	0.4	−0.09 ± 0.09	0.3
Whole model	not applicable		R^2^ = 0.85	*p* < 0.001	R^2^ = 0.51	*p* < 0.001

## Data Availability

The data are available from the corresponding author upon reasonable request.
